# A Metabolome- and Metagenome-Wide Association Network Reveals Microbial Natural Products and Microbial Biotransformation Products from the Human Microbiota

**DOI:** 10.1128/mSystems.00387-19

**Published:** 2019-08-27

**Authors:** Liu Cao, Egor Shcherbin, Hosein Mohimani

**Affiliations:** a Computational Biology Department, School of Computer Science, Carnegie Mellon University, Pittsburgh, Pennsylvania, USA; b National Research University Higher School of Economics, St. Petersburg, Russia; MIT

**Keywords:** natural products, association network, biotransformation, mass spectrometry, metagenomics, microbiome, xenobiotic

## Abstract

The human microbiome consists of thousands of different microbial species, and tens of thousands of bioactive small molecules are associated with them. These associated molecules include the biosynthetic products of microbiota and the products of microbial transformation of host molecules, dietary components, and pharmaceuticals. The existing methods for characterization of these small molecules are currently time consuming and expensive, and they are limited to the cultivable bacteria. Here, we propose a method for detecting microbiota-associated small molecules based on the patterns of cooccurrence of molecular and microbial features across multiple microbiomes. We further map each molecule to the clade in a phylogenetic tree that is responsible for its production/transformation. We applied our proposed method to the tandem mass spectrometry and metagenomics data sets collected by the American Gut Project and to microbiome isolates from cystic fibrosis patients and discovered the genes in the human microbiome responsible for the production of corynomycolenic acid, which serves as a ligand for human T cells and induces a specific immune response against infection. Moreover, our method correctly associated pseudomonas quinolone signals, tyrvalin, and phevalin with their known biosynthetic gene clusters.

**IMPORTANCE** Experimental advances have enabled the acquisition of tandem mass spectrometry and metagenomics sequencing data from tens of thousands of environmental/host-oriented microbial communities. Each of these communities contains hundreds of microbial features (corresponding to microbial species) and thousands of molecular features (corresponding to microbial natural products). However, with the current technology, it is very difficult to identify the microbial species responsible for the production/biotransformation of each molecular feature. Here, we develop association networks, a new approach for identifying the microbial producer/biotransformer of natural products through cooccurrence analysis of metagenomics and mass spectrometry data collected on multiple microbiomes.

## Abstract (precis)

Experimental advances have enabled the acquisition of tandem mass spectrometry and metagenomics sequencing data from tens of thousands of environmental/host-oriented microbial communities. Each of these communities contains hundreds of microbial features (corresponding to microbial species) and thousands of molecular features (corresponding to microbial natural products). However, with the current technology, it is very difficult to identify the microbial species responsible for the production/biotransformation of each molecular feature. Here, we develop association networks, a new approach for identifying the microbial producer/biotransformer of natural products through cooccurrence analysis of metagenomics and mass spectrometry data collected on multiple microbiomes.

## INTRODUCTION

The human microbiome is a complex community of microorganisms, their enzymes, and the molecules they produce/modify. Recent studies show that imbalances in human microbial ecosystems can cause disease. The majority of relationships between the microbiome and disease were discovered through microbiome-wide association studies that link disease to a relative overabundance/underabundance of microbial species using metagenome sequencing data (1, 2). However, these studies fail to determine the molecular mechanism of disease.

Metabolomics studies have shown that among all the molecules in the human metabolome, microbial metabolites are the ones most altered in metabolic and inflammatory disorders (3). These molecules include the biosynthetic products of microbiota (microbial natural products) and the microbial modifications of host, dietary, and drug molecules (microbial biotransformation products) (4).

Currently, the majority of known microbial products and biotransformation products are discovered through the targeted analysis of specific molecules, such as short-chain fatty acids, secondary bile acids, and oral drugs in model systems (e.g., mice with a controlled diet and environment) (5–7). However, these methods do not generalize to complex communities like the human microbiome, where it is impossible to control environmental factors. Moreover, targeted metabolomics analysis cannot detect novel microbial metabolites.

Recent large-scale microbiome data sets, such as the Integrative Human Microbiome Project (iHMP) (8) and the American Gut Project (AGP) (9), collect microbial and molecular abundance profiles over thousands of human microbiota samples, providing us with an unprecedented opportunity to explore the interactions between microorganisms, enzymes, and molecules in complex communities. In these projects, the abundances of tens of thousands of microbial strains/species are measured using microbial marker gene amplicon sequencing and whole-metagenome or metatranscriptome shotgun sequencing (10), and the abundances of tens of thousands of molecules are measured using untargeted liquid chromatography-mass spectrometry (LC-MS) (11). Recently, new methods have been proposed for finding associations between microbial and molecular features through the correlations of their abundance profiles across multiple microbiome samples (12, 13). However, these methods fail to extend to thousands of microbiome samples. In addition, there is no consensus on how to extract features from LC-MS data or what association test should be used.

In this study, we develop an efficient pipeline to discover potential microbial metabolites and microbial biotransformations by building a cooccurrence network of microbes and metabolites using high-throughput LC-MS data and metagenomics data collected over thousands of microbiota samples. Using this strategy, we identify several microbial products and microbial biotransformation products from the human microbiome. Moreover, we develop a new method for computing the false discovery rates (FDR) of the associations and using them to benchmark various metabolomics feature extraction methods and association tests. Furthermore, we develop a new method to detect clade-specific metabolites based on the cooccurrence network and the analysis of a microbial phylogenetic tree.

## RESULTS

### Outline of the pipeline.

**FIG 1:**
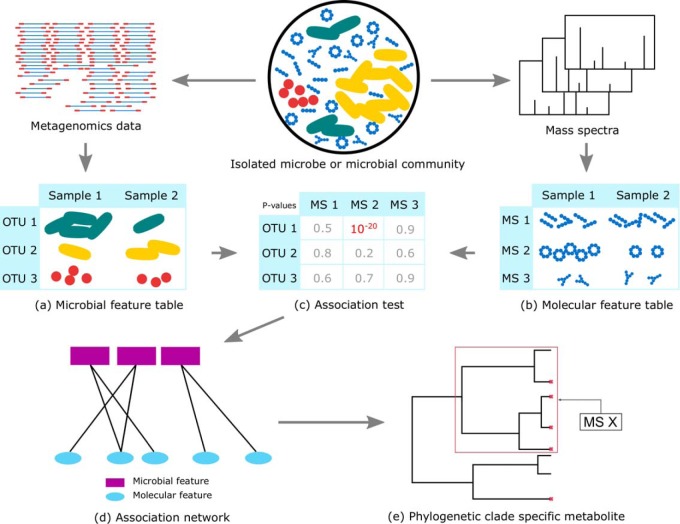
The pipeline includes the following steps: extracting microbial (a) and molecular (b) features from the raw data, searching for pairs of associated features and computing false discovery rates (c), constructing the association network (d), and assigning molecular features to phylogenetic clades (e).

Our pipeline (Fig. 1) includes the following: (a) extracting microbial features, which could be either operational taxonomic units (OTUs) or biosynthetic gene cluster (BGC) families, (b) extracting molecular features, which could be either mass spectrometry (MS) features or tandem mass spectrometry (MS/MS) features, (c) searching for pairs of associated features and computing false discovery rates, (d) constructing the association network, and (e) assigning molecular features to phylogenetic clades.

### Data sets.

The AGP data set consists of LC-MS/MS and 16S rRNA data collected from the human gut microbiomes of 2,125 subjects. For a subset of these samples, shotgun metagenomics data are also available. Optimus extracted 29,567 molecular features from the LC-MS data (MinIntensity = 1,000), and MS-Clustering extracted 74,913 molecular features from the LC-MS/MS data (cosine similarity threshold [τ] = 0.4). We further applied deduplication using an *m/z* threshold of 0.01 and a Fisher’s exact test *P* value threshold of 10^−5^. This decreased the number of molecular features from 29,567 to 18,940 for Optimus and from 74,913 to 73,275 for MS-Clustering. We additionally annotated the extracted molecular features using spectral library search (14) and Dereplicator+ (15). Using the Greengenes Database (16) as the reference, QIIME extracted 11,265 unique OTUs from the AGP data set (MinCount = 0).

The data set for human microbiome isolates from cystic fibrosis patients (HUMAN-CF) consists of tandem mass spectrometry and metagenomics data collected from 243 microbial isolates from cultures of sputum samples from cystic fibrosis patients (Global Natural Product Social Molecular Networking [GNPS] data set MSV000080251). Each sample contains one or a mixture of a few (from 1 to 11) different bacteria. Based on the metagenomics data of HUMAN-CF, Quinn et al. (17) analyzed the association between microbial species and discovered that *Pseudomonas* and Staphylococcus aureus are anticorrelated with Gram-positive anaerobes. In this study, we obtained 23,176 molecular features from LC-MS/MS data (see Materials and Methods for details). We further applied SPAdes (18), antiSMASH (19), and BiG-SCAPE (20) to the shotgun metagenomics data and extracted 18 nonribosomal-peptide BGC families which are present in at least 10 samples.

### Microbial products and biotransformation products.

**FIG 2:**
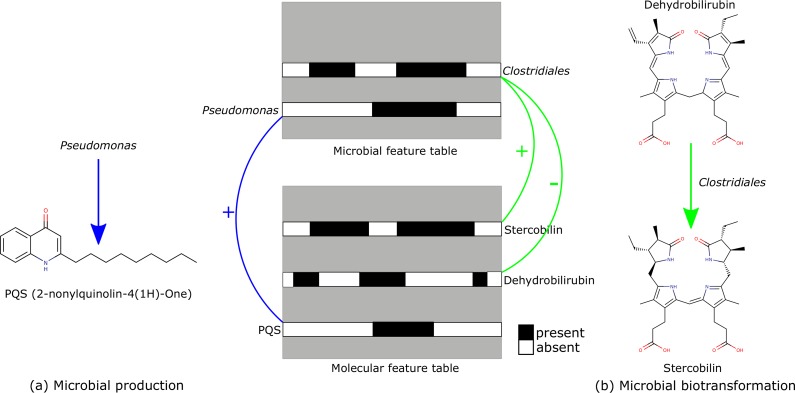
(a) Microbial natural products can be detected as positive correlations between the occurrences of the microbial species and the molecules in the association network. (b) Microbial biotransformation products can be detected as negative correlations between the microbial species and the precursor molecules, along with positive correlations between the microbial species and the product molecules. The feature tables are mock-up data.

Microbial natural products can be detected as positive correlations between the occurrence of the microbial species and the molecules in the association network (Fig. 2a). In addition to the microbial products, the association network also reveals many microbial biotransformation products. Microbial biotransformation products are distinguished by a strong negative correlation between the occurrences of the microbial species and the precursor molecules, along with strong positive correlations between the microbial species and the product molecules (Fig. 2b).

**FIG 3:**
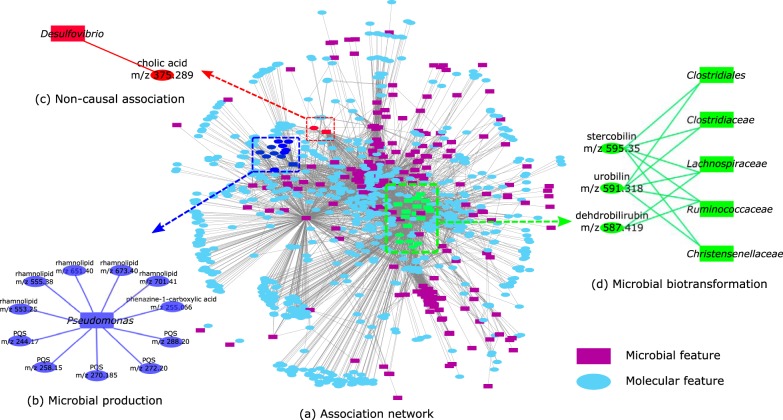
(a) Association network of AGP. (b) *Pseudomonas* bacteria are positively associated with phenazine-1-carboxylic acid, rhamnolipids, and PQS. (c) The correlation between *Desulfovibrio* and cholic acid is noncausal. (d) *Clostridiales* biotransform bile acids. Here, we combined the nodes that represent the same molecules or taxa in the same family.

We applied the association network pipeline to the AGP data set and found 18,623 and 8,178 associations with a *P* value threshold (*P*_Threshold_) of 10^−10^ for the molecular features obtained by Optimus and MS-Clustering, respectively. To explore the power of the association network (Fig. 3) in detecting microbial products and biotransformation products, we further searched the mass spectra against AntiMarin (21), the Dictionary of Natural Products database (22), and the Human Metabolome Database (23) using Dereplicator+ and analyzed the densely connected modules of this network that contained the molecules annotated by Dereplicator+ (Fig. 3).

### Correlating mass spectral data to 16S rRNA data.

At a *P*_Threshold_ of 10^−10^, microbial features from the *Pseudomonas* genus are positively associated with phenazine-1-carboxylic acid (*m/z* 225.07), five rhamnolipids, and five pseudomonas quinolone signals (PQS) (Fig. 3b). Among the 42 rhamnolipids with unique masses produced by *Pseudomonas* (24), 8 are included in the GNPS spectral library. A spectral library search found four of the rhamnolipids in the AGP data set, and two (*m/z* 673.40 and *m/z* 701.41) are significantly associated with *Pseudomonas*. With molecular networking (14, 25), two more rhamnolipids were identified (*m/z* 553.25 and *m/z* 555.38), both of which have a strong association with *Pseudomonas*. *Pseudomonas* is also significantly associated with rhamnolipid B (*m/z* 651.40). Moreover, *Pseudomonas* is positively correlated with compounds from different series of quinolones (26), including 4-hydroxy-2-heptylquinoline-*N*-oxide (*m/z* 258.15), 2-nonyl-4-quinolone (*m/z* 270.19), 2-nonylquinolin-4(1H)-one (*m/z* 272.20), 4-hydroxy-2-nonylquinoline-*N*-oxide (*m/z* 288.20), and 4-hydroxy-2-heptylquinoline (HHQ) (*m/z* 244.169). All of these molecules are known to be produced by Pseudomonas aeruginosa bacteria, playing roles in quorum sensing and virulence (27–29). We further mapped shotgun metagenomics data collected on samples with PQS present against PQS BGC, and we identified 2,472 out of 2,488,704 reads mapped to PQS BGC.

**FIG 4:**
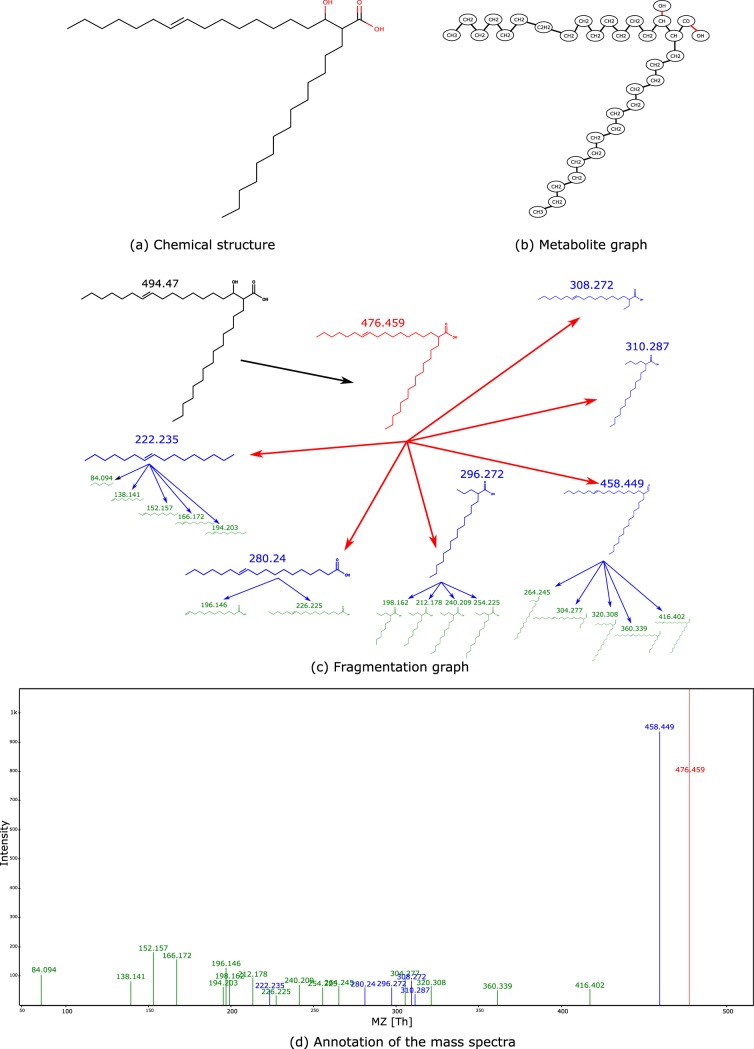
(a) Chemical structure of corynomycolenic acid. (b) Metabolite graph of corynomycolenic acid. (c) Fragmentation graph of corynomycolenic acid. (d) Annotation of the mass spectra of corynomycolenic acid (only explained peaks are shown).

A Corynebacterium kutscheri OTU feature (Greengenes number 13393) is positively correlated with a molecule at *m/z* 495.4 (*P* = 3 · 10^−5^). Dereplicator+ annotated this molecule as corynomycolenic acid (Fig. 4). The BGC for corynomycolic acid, which is a close variant of corynomycolenic acid, has previously been discovered in Corynebacterium diphtheria strain NCTC 13129 (30). The reference genome with a feature closest to this C. kutscheri feature is that of C. kutscheri strain DSM 20755 (31) (99% identical 16S rRNA over 100% coverage), which contains a BGC with high similarity to the corynomycolic acid BGC reported in C. diphtheriae NCTC 13129 (72% identical over 52% coverage).

We also observed a positive correlation between *Desulfovibrio* species and cholic acid (*P* = 10^−13^), which is a human bile acid (Fig. 3c). This is explained by the fact that the *Desulfovibrio* species feed on the sulfur released by deconjugation of taurocholic acids to cholic acid (32). As sulfur is below the dynamic range of mass spectrometers, the association network fails to correlate sulfur with *Desulfovibrio* species. This example shows that some of the detected associations are noncausal.

We observed significant positive correlations between stercobilin (*m/z* 595.35 [*P* = 6 · 10^−29^]), and some of the *Clostridiales*. It is well known that stercobilin and urobilin are the end products of heme catabolism by *Clostridiales* through bilirubin glucuronidase and bilirubin reductase enzymes (33, 34). *Clostridiales* also showed negative correlations with dehydrobilirubin (*m/z* 587.3 [*P* = 10^−30^]) and urobilin (*m/z* 591.35 [*P* = 5 · 10^−26^]), which are the products of bilirubin reductase.

Several species within the *Enterobacteriaceae* showed a negative correlation with cholic acid (*m/z* 409.29 [*P* = 2e−26]) and a positive correlation with 7-oxodeoxycholate (*m/z* 407.28 [*P* = 4e−10]), confirming the evidence that *Enterobacteriaceae* play a role in dehydrogenation of bile acids (35, 36).

We also observed a strong correlation between *Bacillus* species and a steroid hormone with *m/z* 285.18 (*P* = 9 · 10^−24^). *Bacillus* species are known to biotransform steroids (37).

In addition, we observed a negative correlation between *Oxalobacteraceae* and phenylalanine (*m/z* 165.08 [*P* = 6 · 10^−11^]) and *n*-acetylphenylalanine (*m/z* 207.12 [*P* = 3 · 10^−13^]). In fact, phenylalanine and *n*-acetylphenylalanine were not detectable in any of the subjects where *Oxalobacteraceae* were present. *Oxalobacteraceae* species are shown to be capable of consuming phenylalanine as a carbon source (38).

*Clostridiales* species showed negative correlations with phenylalanine (*m/z* 165.08 [*P* = 2 · 10^−15^]), tryptophan (*m/z* 206.07 [*P* = 10^−27^]), dihydroxyphenylacetic acid (*m/z* 153.056 [*P* = 3 · 10^−11^]), and tyrosine (*m/z* 182.08 [*P* = 5 · 10^−13^]) and a positive correlation with indolepropionate (*m/z* 190.018 [*P* = 8 · 10^−11^]). *Clostridiales* is known to biotransform the phenyl residue in these molecules (39).

### Correlating mass spectral data to BGC families.

**FIG 5:**
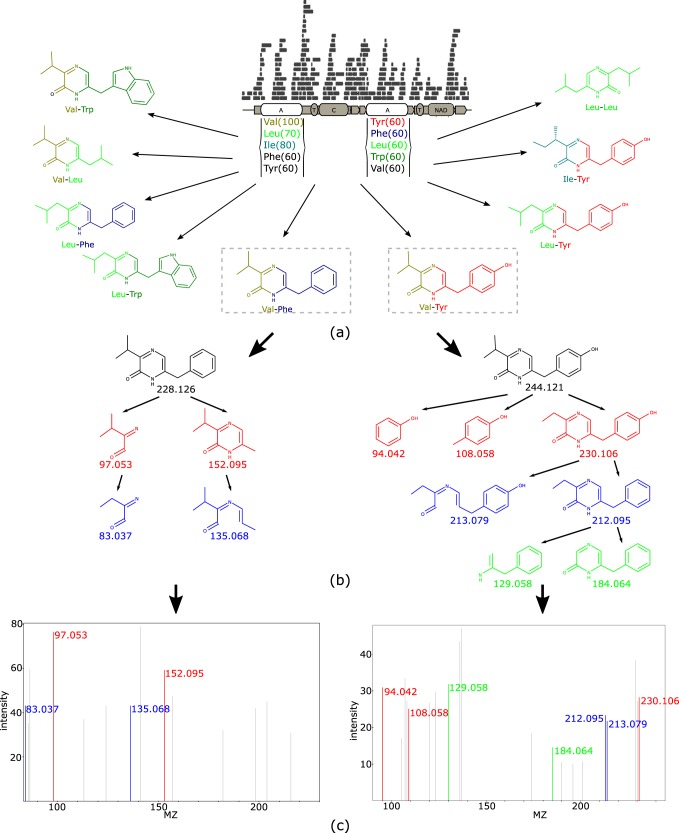
BGC of phevalin. (a) Putative nonribosomal peptide synthetase (NRP) BGC discovered by antiSMASH. This BGC contains two adenylation domains (A), two thiolation domains (T), one condensation domain (C), and one NAD binding domain (NAD). Under each adenylation domain are the associated amino acids and scores predicted by NRPSPredictor. The greater the score, the greater the likelihood that the amino acid will be recognized by the adenylation domain. The surrounding structures are the putative molecules that can be produced by the BGC. (b) Fragmentation tree of Val-Phe (phevalin) and Val-Tyr (tyrvalin) given by Dereplicator+. (c) Mass spectral annotations given by Dereplicator+.

In the HUMAN-CF data set, we correlated BGC families with molecular features and discovered an interesting BGC family containing two adenylation domains, two thiolation domains, one condensation domain, and one NAD binding domain (Fig. 5a) that was positively correlated with two molecular features (*m/z* 229.135 [*P* = 4.05 · 10^−16^] and *m/z* 245.125 [*P* = 1.98 · 10^−9^]). Dereplicator+ annotated these two features as phevalin (score of 4) and tyrvalin (score of 7). These annotations matched the adenylation specificities of the corresponding domains (Fig. 5). BLAST results suggest that this BGC family contains the aureusimine nonribosomal peptide synthetase from Staphylococcus aureus (100% coverage and 99.46% identity), which is known for the synthesis of phevalin and tyrvalin (40). 16S rRNA sequencing results show that Staphylococcus aureus is widely present in the HUMAN-CF data set (17).

### Discovering a corynomycolenic acid BGC.

**FIG 6:**
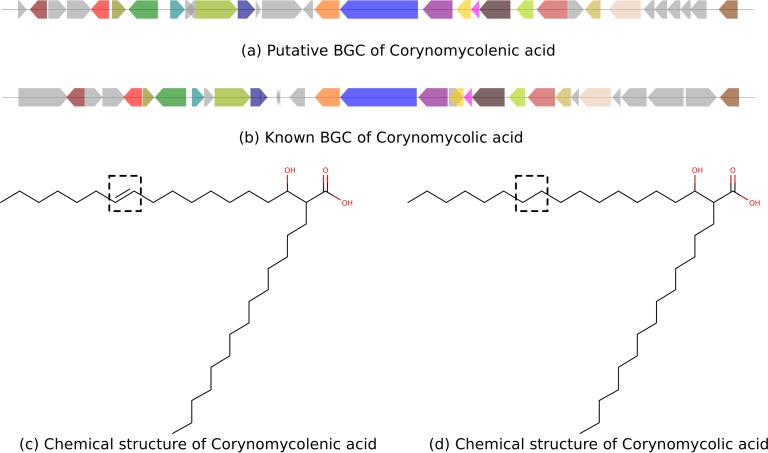
(a) Putative BGC of corynomycolenic acid in Corynebacterium kuscheri strain DSM 20755. (b) Known BGC of corynomycolic acid in Corynebacterium diphtheria strain NCTC 13129. Genes annotated with the same function in the two BGCs are in the same color. Genes in gray are unique genes of the two BGCs. (c) Chemical structure of corynomycolenic acid. (d) Chemical structure of corynomycolic acid. The structural difference between the two molecules is highlighted in black boxes.

We further investigated the genes responsible for the production of corynomycolenic acid in the human microbiota. Corynomycolenic acid is a member of the mycolic acid family with immunomodulatory activities that is produced by *Corynebacterium* and *Mycobacterium* species (41–44). These molecules are ligands of human T cells, prompting specific immune responses. Mining the genome of C. kutscheri DSM 20755 revealed a BGC that contains all the necessary biosynthetic enzymes for the production of corynomycolenic acid (Table 1, Fig. 6). Moreover, we highlight the different genes of the two BGCs which are potentially responsible for the structural difference between the molecules from the two species (Table 2).

**TABLE 1:** Shared genes of the corynomycolic acid BGC from Corynebacterium diphtheria NCTC 13129 and the putative corynomycolenic acid BGC from Corynebacterium kutscheri DSM 20755^*a*^

Shared gene in BGC from:								COG	Protein function^*b*^
*C. diphtheria* NCTC 13129				*C. kutscheri* DSM 20755					
Position		Strand	Gene^*b*^	Position		Strand	Gene^*b*^		
Start	End			Start	End				
4169	3030	−		1837	776	−		COG1835	Acyltransferase
7716	6562	−	*pimB* [H]	5690	4560	−	*rfaB* [C]	COG0438	Mannosyltransferase/glycosyltransferase
7758	8492	+	*ubiE* [C]	5875	6735	+		COG0500	Methyltransferase
10460	8517	−	*pckG* [H]	8719	6896	−	*pckG* [H]	COG1274	Phosphoenolpyruvate carboxykinase
10812	11591	+	*trmB*	9478	10401	+	*trmB* [H]	COG0220	tRNA methyltransferase
12223	14463	+	*mmpL3* [H]	11071	13662	+	*mmpL3* [H]	COG2409	Putative membrane protein
14450	15508	+		13666	14745	+		COG0392	Membrane protein
19989	18439	−	*pccB* [H]	19980	18418	−	*accD5* [H]		Propionyl-CoA carboxylase beta chain
24761	20001	−	*ppsA* [H]	24845	20001	−	*ppsA* [H]	COG3321	Polyketide synthase
26674	24860	−	*fadD32* [H]	26987	25143	−	*fadD32* [H]	COG0318	Long-chain fatty acid–AMP ligase
27660	26749	−		28128	27214	−			Cutinase
28181	27666	−		28649	28134	−			Hypothetical protein DIP
30205	28181	−	*csp1* [H]	30577	28646	−	*csp1* [H]	COG0627	Protein PS1 [H]
31486	30458	−	*csp1* [H]	32001	30934	−	*fbpC* [H]	COG0627	Protein PS1 [H]/antigen 85-C [H]
33329	31641	−		34132	32198	−			Transmembrane protein
34315	33338	−		36163	35192	−		COG0382	Protein y4nM [H]
36791	34806	−	*glfT2* [H]	38653	36674	−	*glfT2* [H]		UDP-galactofuranosyl transferase
44742	43552	−	*rfbD* [H]	44664	43483	−	*rfbD* [H]	COG0562	UDP-galactopyranose mutase

a The genes were annotated by using BASys (45).

b Results given by similarity search in BASys are indicated as follows: [H], homology to a SwissProt entry; [C], homology to a CCDB entry.

**TABLE 2:** Unique genes of the corynomycolic acid BGC from Corynebacterium diphtheria NCTC 13129 and the putative corynomycolenic acid BGC from Corynebacterium kutscheri DSM 20755^*a*^

Source of BGC	Gene position		Strand	Gene^*b*^	COG	Function
	Start	End				
*C. diphtheria* NCTC 13129	61	3138	+			Coagulation factor 5/8-type domain-containing protein
	4176	5276	+			Hypothetical protein Cauri
	5267	6679	+			Integral membrane protein
	11576	12208	+			Hypothetical protein
	15103	15002	−			Hypothetical protein
	16160	16059	−			Hypothetical protein
	16147	16251	+			Hypothetical protein
	16296	16153	−			Hypothetical protein
	17827	16859	−			Cell wall surface anchor family protein
	26737	27669	+			Hypothetical protein
	34806	34312	−		COG0671	Membrane-associated phospholipid phosphatase
	37391	36876	−	*ybjG* [C]	COG0671	PAP2 superfamily protein
	39009	37432	−	*gbsA* [H]	COG1012	Betaine aldehyde dehydrogenase
	41301	39076	−	*betT* [H]	COG1292	High-affinity choline transport protein
	41438	43366	+	*betA* [H]	COG2303	Choline dehydrogenase

*C. kutscheri* DSM 20755	41	601	+			Hypothetical CgR protein
	1923	3059	+			Hypothetical protein A
	3084	4592	+			Hypothetical protein A
	10402	11067	+			Hypothetical
	11081	10443	−			Hypothetical protein
	14777	15109	+			Hypothetical protein Cauri
	15170	17683	+	*pepN* [H]	COG0308	Aminopeptidase N
	18337	17705	−	*pcp* [H]	COG2039	Pyrrolidone-carboxylate peptidase
	34155	35132	+			Hypothetical Protein
	39510	38839	−	*ideR* [H]	COG1321	Iron-dependent repressor
	40294	39497	−	*znuB* [C]	COG1108	29-kDa membrane protein in *fimA* 5′ region
	41112	40291	−	*yfeC* [H]	COG1108	Chelated iron transport system membrane protein
	41752	41099	−	*mntB* [H]	COG1121	Manganese transport system ATP-binding protein
	42741	41713	−	*mntA* [H]	COG0803	Manganese-binding lipoprotein

a The genes were annotated by using BASys (45).

b Results given by similarity search in BASys are indicated as follows: [H], homology to a SwissProt entry; [C], homology to a CCDB entry.

### Assigning molecular features to the corresponding phylogenetic clades.

**FIG 7:**
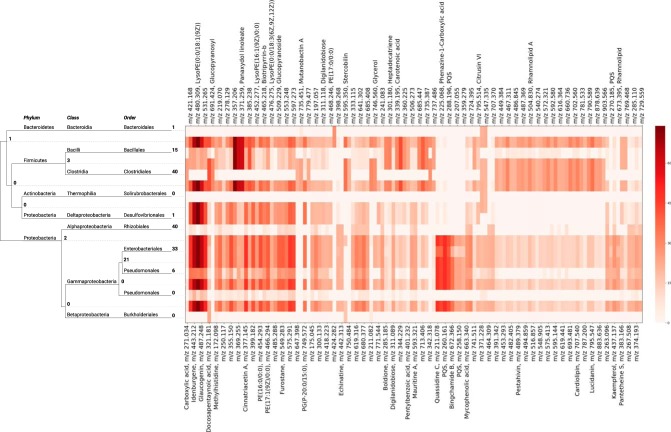
Assigning the molecular features that are positively associated with the microbial features at a *P* value threshold of 10^−20^ to the phylogenetic tree. The tree is trimmed to the taxonomic-order level. Numbers in boldface show the counts of molecules assigned to the corresponding clades. Heatmap shows −log_10_(*P*), where *P* is the minimal *P* value between the molecule and an OTU within the clade. Dereplicator+ molecular annotations for the known molecules are shown. The molecular features were extracted by MSClustering based on tandem mass spectral data and annotated by spectral library search and Dereplicator+ (level 2 and 4 metabolite identification) (46).

We assigned the molecular features to the clades in the phylogenetic tree with which they were significantly associated. For this analysis, we used the Greengenes phylogenetic tree, which was pruned to keep only the OTUs that were associated with at least one metabolite. At a *P* value threshold of 10^−10^, 550 of the MS-Clustering features were mapped to 872 OTUs in the phylogenetic tree. Figure 7 demonstrates molecular features assigned to different clades at a *P* value threshold of 10^−20^.

### Benchmarking.

**FIG 8:**
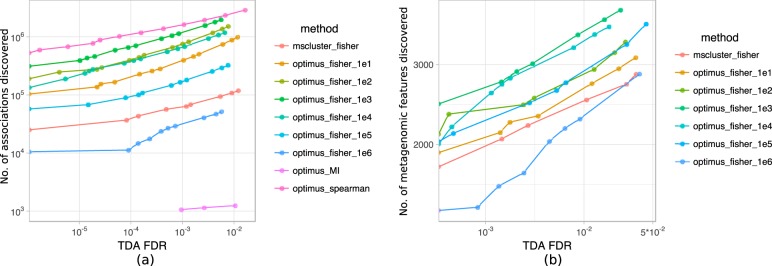
Benchmarking various feature extraction methods and association tests. Different methods are compared based on the number of associations discovered (a) and the number of unique metagenomic features associated with a molecular feature (b) at different false discovery rate thresholds. Here, we benchmark MS-Clustering and Optimus (binarized abundance with thresholds 10, 10^2^, …, 10^6^) with Fisher’s exact test association and Optimus (continuous abundance) with Pearson’s correlation test association, Spearman’s rank correlation test association, and mutual information criterion. In the case of Pearson’s correlation, no association was discovered at a false discovery rate of 0.01.

**FIG 9:**
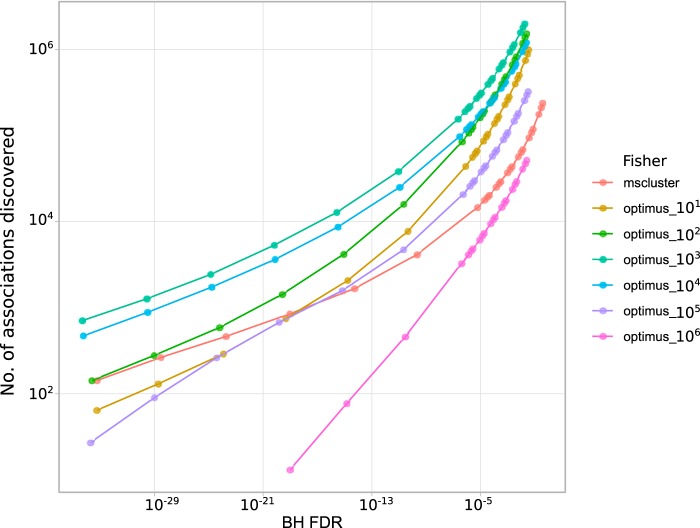
Benchmarking MS-Clustering and Optimus (binarized abundance with thresholds 10, 10^2^, …, 10^6^) with Fisher’s exact test association. Data on the *x* axis represent false discovery rates estimated by the Benjamini-Hochberg procedure. Data on the *y* axis represent the numbers of metabolite-microbe associations discovered.

We benchmarked various feature extraction methods with various parameters by comparing the numbers of identifications at different false discovery rates. Moreover, we benchmarked four different techniques for estimating the associations between molecular and microbial features. These techniques include Fisher’s exact test (for binary data), Pearson’s correlation test, Spearman’s correlation test, and the mutual information criterion. Our results show that Optimus and Spearman’s correlation are the best feature extraction and association methods (Fig. 8 and 9).

## DISCUSSION

Recent experimental advances have enabled the acquisition of tandem mass spectrometry and shotgun metagenomics data from tens of thousands of environmental/host-oriented microbial communities through large-scale projects, including the American Gut Project and the Integrative Human Microbiome Project. Metagenome-mining studies have revealed thousands of biosynthetic enzymes with uncharacterized substrates/products from these data sets. Moreover, metabolomics studies have revealed signals for hundreds of thousands of bioactive small molecules in the mass spectral data sets.

While these data sets represent a gold mine for discovering small molecules associated with the microbiota, manual analysis of billions of mass spectra in these data sets is infeasible, and new computational approaches are needed to integrate the large-scale metagenomics and tandem mass spectrometry data for systematic discovery of the unknown small-molecule products of the biosynthetic enzymes. In this regard, the following three questions need to be addressed. (i) Is the molecular feature associated with the microbiota? If so, which microbial species is it associated with? (ii) Which biosynthetic enzyme within the microbial species is it associated with? (iii) What is the chemical structure of the molecule?

In this article, we developed a method for addressing the first question. Our method detects microbial natural products and microbial biotransformation products through a comparative analysis of the molecular and microbial features across multiple microbiomes. In the case of corynomycolenic acid, we further used genome mining to assign the molecule to its BGC within the genome of its microbial producer. While identification of the biosynthetic enzymes responsible for corynomycolenic acid production provides a proof of concept, novel computational methods are needed for systematic characterization of the products of the microbial biosynthetic enzymes through the association network approach.

The association network detects pairwise interactions between the molecular and microbial features across thousands of microbiomes. While this method is capable of discovering microbial natural products and microbial biotransformation products, interactions that involve multiple sequential biotransformations/complex pathways cannot be handled. Moreover, many of the interactions retrieved by this method are noncausal correlations. For example, the association network finds correlating features that are caused by a confounding factor. While this results in a denser network with noncausal edges, in some scenarios, these noncausal edges can lead to the discovery of causal interactions that were missed by the network.

Currently, the association network approach is based on the use of Fisher’s exact test *P* values, which assumes different samples are independent. While the independence assumption is natural for data sets such as that of the American Gut Project, collected from distinct individuals, confounders like health status could increase the false discovery rate. The association network approach is the first step toward detecting the complex interactions between microbial and molecular features through the comparative analysis of thousands of microbiome samples.

In addition to linking BGCs to molecules, other potential applications of association networks include detection of carbon sources depleted by microorganisms, identifying biomarkers for drug metabolism, linking microbial enzymes to xenobiotic metabolism, and identifying the role of microbial metabolites in disease. Association networks provide an untargeted approach for generating/testing various hypotheses about the causal relationships between the molecules and microbes in complex communities.

## MATERIALS AND METHODS

### Definitions.

Consider a set of microbial community samples (Samples), a set of molecular features (Molecules), and a set of microbial features (Microbes). Here, each molecular feature is the abundance of a specific molecule (binary or continuous), and each microbial feature is the abundance of a specific microbe. Every feature *X* is characterized by a subset of samples that *X* is present in, as follows: Samples_*X*_={*S*∈Samples|*X* is present in *S*}. Here, *S* represents a sample.

### Inputs.

The inputs to our pipeline are the untargeted mass spectrometry data and metagenomics data collected on a set of microbiome samples.

### Main pipeline.

The association network pipeline consists of the following steps.

(i) For microbial feature extraction, QIIME (47) is used to extract and quantify the operational taxonomic units (OTUs) from the 16S rRNA sequencing data. The QIIME output is the OTUCount matrix, where OTUCount(*A*, *S*) is the number of times an OTU *A* is observed in a sample *S*. For each OTU *A*, we define Samples_*A*_={*S*|OTUCount(*A,S*)>MinCount} for a threshold MinCount.

When shotgun metagenomics data are available, we can quantify BGC families on top of OTUs. First, we apply SPAdes (18) to metagenomics data to obtain genome assemblies. Second, we apply antiSMASH (19) to the genome assemblies to extract putative BGCs. Third, we use BiG-SCAPE (20) to cluster similar BGCs into BGC families, resulting in an absence-presence table of the BGC families in each sample. We exclude from analysis rare BGC families that are present in less than 10 samples.

(ii) For molecular feature extraction, molecular features from the liquid chromatography-mass spectrometry (LC-MS) data are first extracted and quantified using the feature extraction algorithm Optimus (48). Optimus outputs the FeatureIntensity matrix, where FeatureIntensity(*X*, *S*) is the intensity of a feature *X* in a sample *S*. We then select a threshold MinIntensity, and for every feature *X*, we define Samples_*X*_={*S*|FeatureIntensity(*X,S*)>MinIntensity}. We further remove molecular features that are present in less than two samples. When LC-MS/MS data are available, we extract molecular features using the MS-Clustering algorithm (49). Since the LC-MS/MS data are more suitable for molecular-feature annotation, we use MS-Clustering as the molecular-feature extraction method when analyzing the AGP and HUMAN-CF data sets.

We also construct a set of decoy molecular features, DecoyMolecules (Fig. 1b). These decoy molecules are used to estimate the FDR. The set DecoyMolecules is created as follows: for every feature *X*∈Molecules, we construct a decoy feature *X_d_*, with Samples_*X*_*d*__ being a randomly chosen subset of Samples with size |Samples_*X*_|.

(iii) To perform the association test, we then search for pairwise associations between Molecules and Microbes (Fig. 1c). More specifically, we look for pairs (*X*, *A*) consisting of a molecular feature *X* and a microbial feature *A* that have a statistically significant correlation in their patterns of occurrence.

Given two features *X* and *A*, to detect whether *X* and *A* are cooccurring, we consider the null hypothesis that the events “*X* is present in a sample” and “*A* is present in a sample” are independent. A statistically significant correlation in the patterns of occurrence of *X* and *A* is detected if the *P* value of Fisher’s exact test, denoted *P*_Value_(*X*, *Y*), is lower than the selected threshold *P*_Threshold_, and the null hypothesis is rejected.

While there are other techniques for computing the associations between the molecular and microbial features, including Pearson’s correlation, Spearman’s correlation, and mutual information criterion, in this section, we focus on the Fisher’s exact test method.

For the multiple-hypothesis testing, we compute the FDR using the target-decoy approach (TDA) (50). We first search for the associations between DecoyMolecules and Microbes and then estimate the FDR as |DecoyAssociations| / |RealAssociations|, where DecoyAssociations and RealAssociations are the sets of association pairs found in decoy and target data sets. We also use the Benjamini-Hochberg (BH) procedure for estimating the FDR.

(iv) To build the associations network, we further construct a bipartite network where the vertices are the molecular and microbial features and there is an edge between two vertices if the corresponding features are associated (Fig. 1d).

(v) We also report the associations between the molecular features and the groups of related microbial features by assigning molecular features to the clades in the phylogenetic tree that are potentially responsible for their production/biotransformation (Fig. 1e). Note that here, assignment of a molecule to a phylogenetic clade does not necessarily mean that the molecule is produced by those species. For example, those species might play a role in biotransformation of the molecule.

Given a phylogenetic tree *T* and a molecular feature *X*, we first mark all the microbial features that are positively correlated to *X* and count the number of marked features in every clade. Then, we select the minimal clade that has at least *P* percent (*P = *80) of features marked. If the selected clade is a proper subset of the whole tree, we assign *X* to this clade. We perform the steps described for every molecular feature, and for each clade, we report the set of molecular features that are assigned to it.

### Deduplication of molecular features.

Feature extraction methods usually report redundant features, i.e., each single molecule is reported as multiple features with similar *m/z* values. Such features are called “duplicates.” The process of finding all groups of duplicate features and merging them into unique features is called “deduplication.” We apply deduplication to remove the redundancy in the molecular features.

We consider a pair of molecular features to be duplicates if they have similar *m/z* values and a statistically significant correlation in their patterns of occurrence. Then, we build a graph in which molecular features are nodes and every putative pair of duplicates is connected by an edge. The connected components of the resulting graph are the groups of duplicate features. For the *i*-th group DuplicatesGroup*_i_*, a new consensus feature *Y_i_* is constructed with the *m/z* being the average *m/z* of all the features in DuplicatesGroup*_i_*, and Samples_*Y*_*i*__ is defined as the union

$$\underset{X\in {\text{DuplicatesGroup}}_{i}}{\cup }{\text{Samples}}_{X}.$$

### Benchmarking.

Molecular-feature extraction consists of identification and quantification of the peaks across multiple LC-MS runs and is a fundamental step in proteomics and metabolomics. Although many tools for molecular-feature extraction have been proposed, it is not clear which one is more accurate. Moreover, it is not clear how to adjust the parameters in various feature extraction methods.

Here, we describe an approach to compare the various feature extraction methods in the microbiome-wide correlation studies. Given a set of microbial features and several feature extraction methods with various sets of molecular features, we apply the pairwise association pipeline to these sets to identify the method and the parameter settings that result in the highest number of pairs of cooccurring features discovered at a certain FDR level. To avoid bias toward methods that report higher numbers of molecular features, we also compare the numbers of discovered microbial features in these pairs. The FDR is estimated by the target-decoy approach (TDA) and the Benjamini-Hochberg procedure. Four different association tests are benchmarked, including Fisher’s exact test, Pearson’s correlation test, Spearman’s rank correlation test, and the mutual information criterion.

### Data availability.

The association networks computer code is available on GitHub at https://github.com/mohimanilab/AssociationNetworks.
